# Inheritance of coat colour in the cane Corso Italiano dog

**DOI:** 10.1186/s12863-019-0731-2

**Published:** 2019-03-04

**Authors:** Evžen Korec, Matyáš Hančl, Marie Bydžovská, Ondřej Chalupa, Jana Korcová

**Affiliations:** Department of Genetics, ZOO Tábor, Dukelských hrdinů 19, 17000 Prague 7, Czech Republic

**Keywords:** Cane Corso Italiano, Coat colour, Dog breed, Genetics, Sex chromosome, Inheritance

## Abstract

**Background:**

The inheritance of different coat colours in the Cane Corso Italiano dog has not been described thus far. We analysed data from 23,271 dogs and bitches using the Cane Corso Italiano Pedigree Database. We are describing for the first time the coat colour segregation ratios in Cane Corso Italiano offspring arising from crosses between parents of all possible coat colour combinations.

**Results:**

Segregation ratios that do not follow a Mendelian pattern suggest that additional genes are active in the determination of coat colour. Segregation ratios of offspring produced by parental crossing (male colour A x female colour B) were compared with the ratios of offspring produced by reciprocal crossing (male colour B x female colour A) in all possible coat colour combinations.

Most of the segregation ratios were the same, but some segregation ratios in reciprocal crosses differed. This result suggests that at least one gene responsible for coat colour is located on a sex chromosome. The sex ratio was analysed in the offspring of all colour groups. A ratio of 1:1 was not confirmed in 8 colour groups by the chi-square test.

**Conclusions:**

We described for the first time coat colour segregation ratios in Cane Corso Italiano dogs. Furthermore, we present the hypothesis that at least one gene responsible for coat colour is located on a sex chromosome.

## Background

Coat colour variations in different dog breeds have been a point of interest to breeders and researchers for a long time. The first papers concerning the coat colours of dogs appeared at the beginning of the twentieth century [1].

Little [2] and Winge [3] both published books suggesting a number of genes that could explain the inheritance of coat colour. Subsequently, many genes responsible for a dog’s coat colour have been discovered and described during the past two decades [4–20]. However, a widely accepted nomenclature for gene names and symbols in domestic dogs does not exist; some genes were named historically by the scientists who first described them, and some genes were named according to their role in humans or a model organism. This was confusing for breeders as well as for scientists. A review by Kaelin and Barsh [21] summarized the issue of dog coat colour genetics, introduced a modified version of the historical nomenclature and identified 12 coat colour loci in dogs. However, biological mechanisms are still unknown for some coat colour phenotypes.

**Fig. 1:**
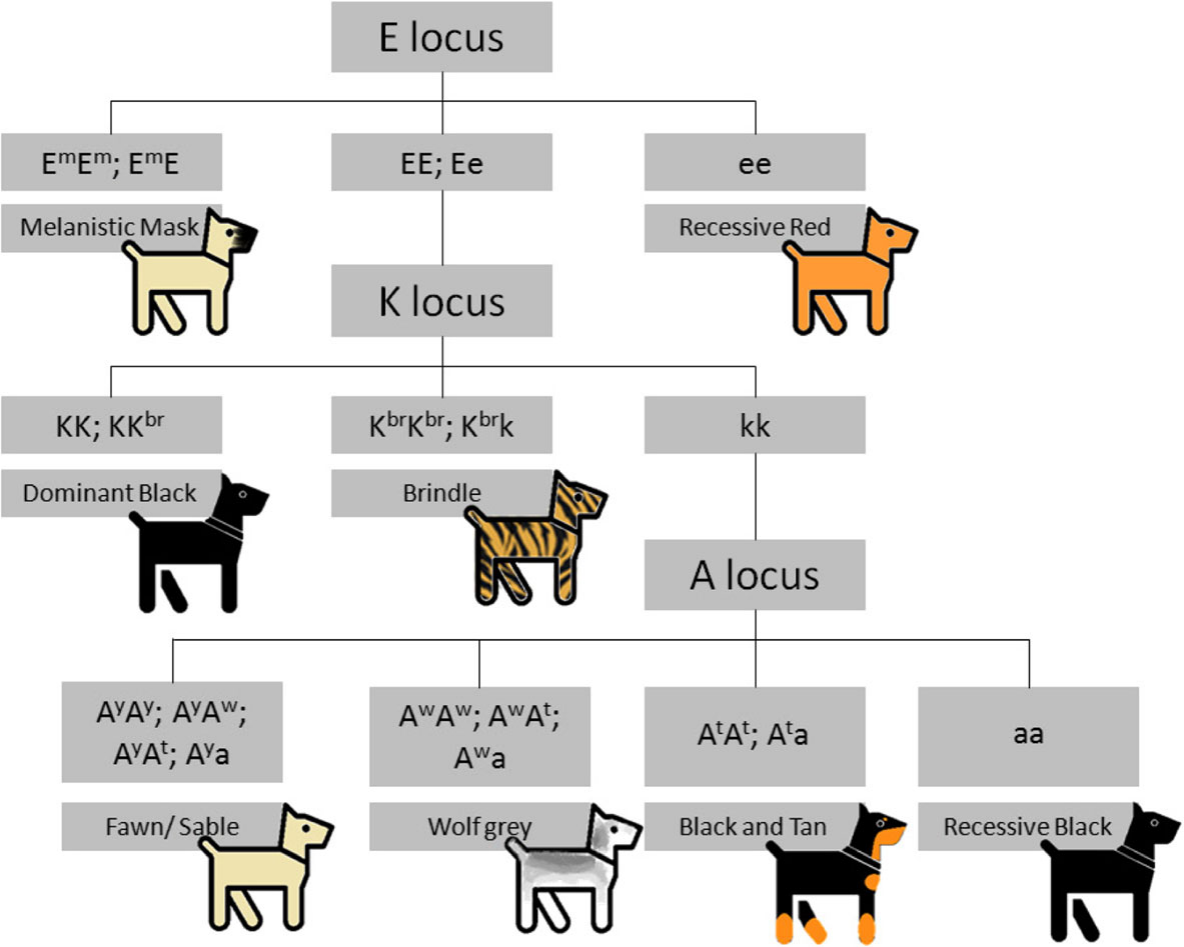
Inheritance of the basic coat colour loci. Locus E affects the distribution of eumelanin and pheomelanin. In dogs, there are three alleles with specific dominance (E^m^ > E > e). E^m^ is responsible for melanistic mask and e is responsible for recessive red. The K locus is hypostatic for the E locus, and there are also three alleles with a specific hierarchy (K > K^br^ > k). The K allele is responsible for dominant black and K^br^ is responsible for brindle colour. Locus A has four alleles, and the dominance of these alleles is A^y^ > A^w^ > A^t^ > a. Allele A^y^ is responsible for fawn or sable colour. Allele A^w^ represents wild colouration, which is ancestral. Allele A^t^ represents black and tan or saddle and tan colouration, and allele a is responsible for recessive black. Alleles E and k are wild-type, and coat colour is under the control of hypostatic loci

Melanocortin 1 receptor (MC1R) was the first gene discovered by a molecular genetic analysis in dogs [22]. This gene can be identified with allelic locus E, and mutations in this gene commonly result in a distribution of eumelanin and pheomelanin. An important breakthrough was the discovery that pigment type-switching (black eumelanin and yellow pheomelanin) is controlled by three genes in dogs: Melanocortin 1 receptor (MC1R), Agouti signalling protein (ASIP) and β–Defensin 103 (CBD103) [23, 24]. These genes are associated with characteristic allelic influence (locus E, locus K and locus A) for a dog’s coat colour. Allelic dominance and the hierarchy of individual loci are summarized in Fig. 1 according to Kaelin and Barsh’s review [21].

**Fig. 2:**
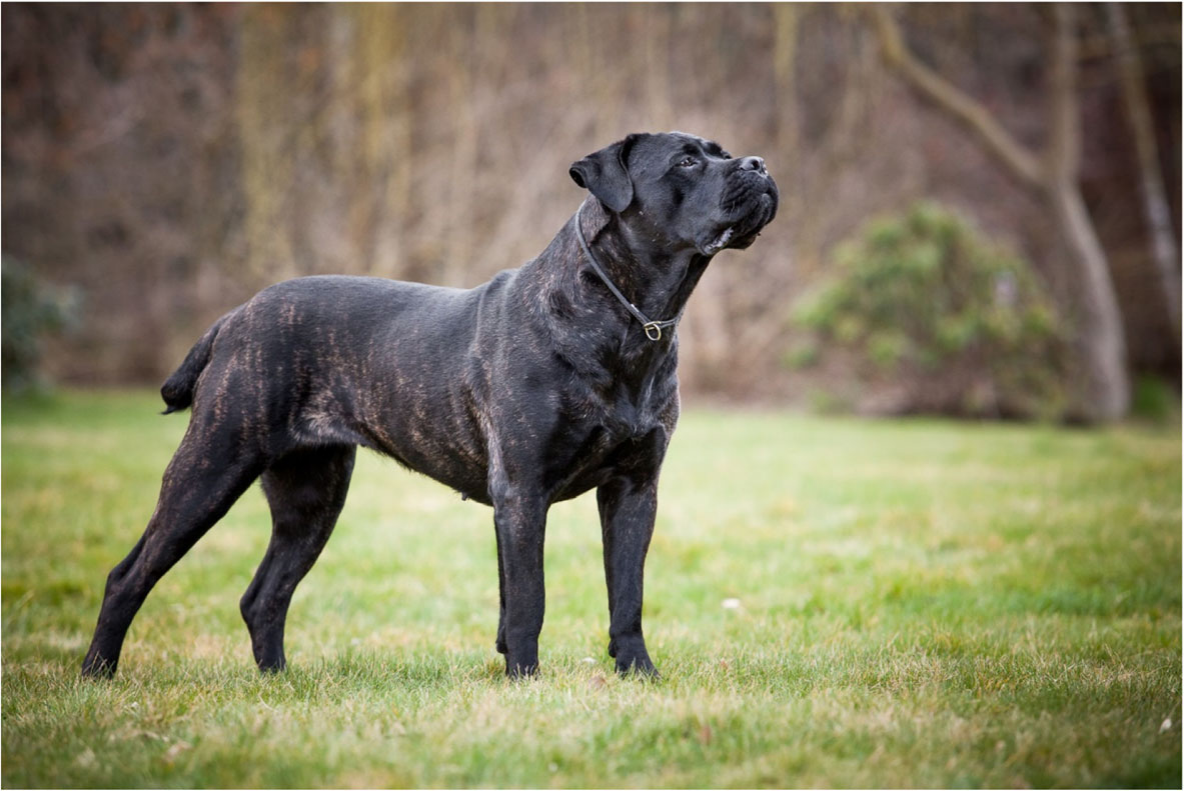
Photograph of a Cane Corso Italiano dog (Koleta Atison, Evžen Korec’s archive)

The Cane Corso Italiano (Fig. 2) is a breed with a great history; however the breed went almost extinct in the mid-twentieth century. Since then, the population of Cane Corsos has grown thanks to selective breeding. This breed was successfully accepted by the Italian Kennel Club (ENCI) in 1994 and was fully recognized by the Federation Cynologique Internationale (FCI) in 2007.

The results of the first research on this breed were described by Korec et al. [25]. In most breeds, a relatively narrow range of colours are allowed within the standard, and in some breeds, all dogs have the same colour pattern. In the Cane Corso Italiano breed, the FCI standard allowed the following colours: black, black brindle, brindle, fawn, grey and grey brindle. The inheritance patterns of different coat colours in the Cane Corso Italiano has not been described so far.

## Methods

### Data collection

All data regarding the Cane Corso Italiano breed were obtained from the Cane Corso Italiano Pedigree Database [26] (www.canecorsopedigree.com/). Data collection was focused on the coat colour and sex of 82,169 individuals. Individuals without a complete data set were excluded from the analyses (71.7%). Only data of litters with complete information about parents and offspring (28.3%) were used for further analysis.

### Statistical methods

For the statistical analysis, we used data from 23,271 individuals (dogs, bitches and offspring). The chi-square test [27] was used for statistical confirmation of segregation ratios. A *p*-value < 0.05 was considered statistically significant.

## Results

**Fig. 3:**
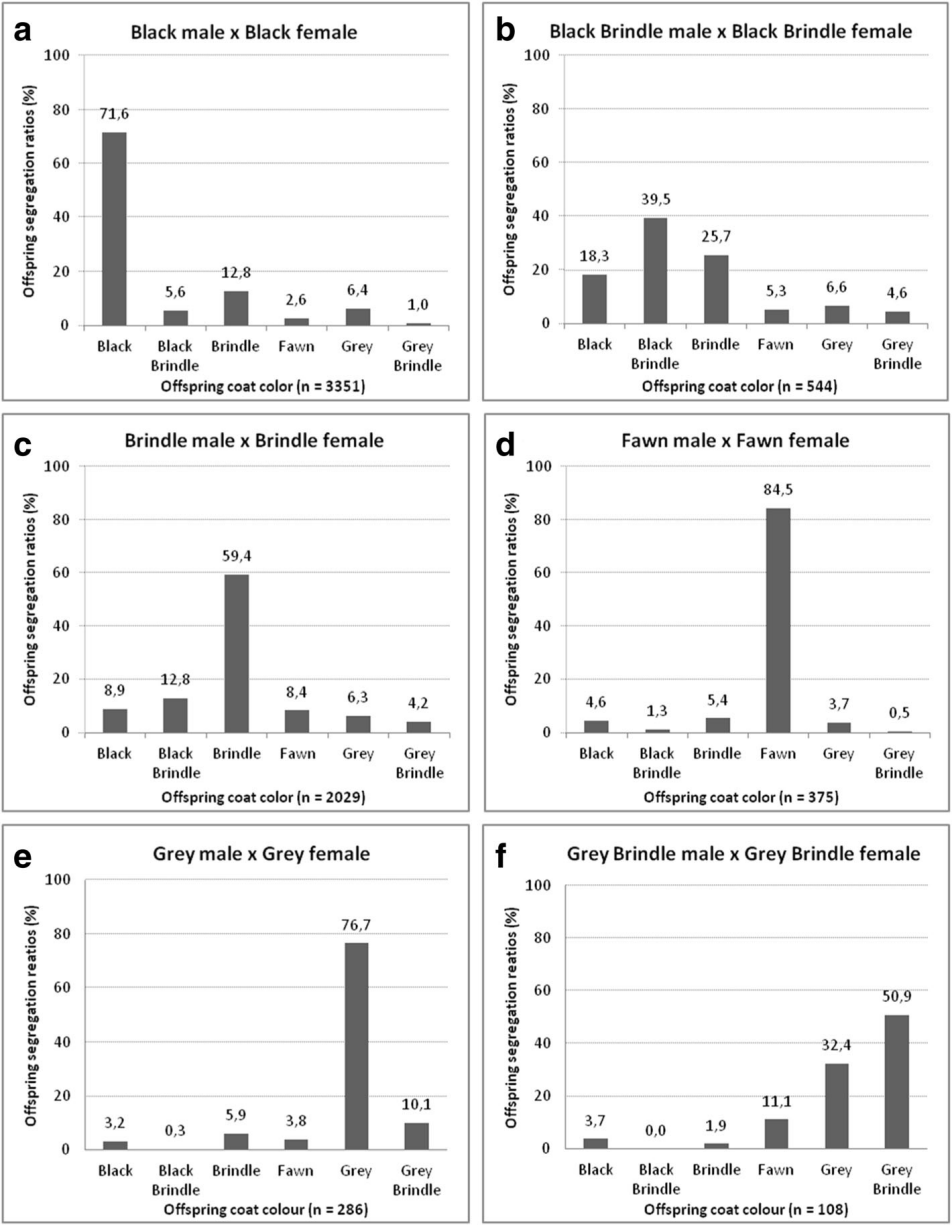
Male and female crossing of the same colour in the Cane Corso breed. **a** crossing two black parents. **b** crossing two black brindle parents. **c** crossing two brindle parents. **d** crossing two fawn parents. **e** crossing two grey parents. **f** crossing two brey brindle parents

The main aim of this study was to identify the principles of inheritance of coat colours in Cane Corso Italiano breed. First, we attempted to verify whether the principles of Mendelian inheritance of the coat colour could be applied. Crosses between males and females of the same colour are summarized in Fig. 3.

Black is the most frequent coat colour in the Cane Corso breed. The segregation ratio of the offspring produced by crossing two black parents is summarized in Fig. 3a. Of the offspring, 71.6% were black, and 28.4% were other colours.

Crossing two black brindle parents resulted in segregation of the offspring, where only 39.5% of the offspring were black brindles, 25.7% were brindles and 34.8% had other colours (Fig. 3b). Crossing two brindle parents resulted in 59.4% brindle offspring and 40.6% other-colour offspring (Fig. 3c). Crossing two fawn parents resulted in 84.5% fawn offspring and 15.5% other-colour offspring (Fig. 3d). Crossing two grey parents resulted in 76.7% grey offspring and 23.3% other-colour offspring (Fig. 3e), and grey brindle parents produced 50.9% grey brindle offspring and 49.1% other colour offspring (Fig. 3f).

These results suggest that the inheritance of coat colour is much more complicated and that additional genes are involved. The segregation ratios of offspring produced by the parental crossing of all possible colour variations suggest that at least one gene responsible for coat colour is located on a sex chromosome. To verify this hypothesis, the sex ratio was analysed in offspring of all colour groups using statistically significant data. A ratio of 50% males and 50% females should be found if all genes responsible for coat colour are located only on autosomes. A ratio of 1:1 was found in most colour groups and was confirmed by the chi-square test. A sex ratio of 1:1 was not confirmed in 8 offspring colour groups by the chi-square test. These offspring colour groups are summarized in Table 1.

**Table 1:** Offspring colour groups with sex ratios different than 1:1

Parents		Offspring			Chi - square test
Male colour	Female colour	Colour	Number of males	Number of females	*p*-value
Brindle	Grey brindle	Brindle	90	55	0.0037
Black	Brindle	Black	524	615	0.0070
Black	Black	Black	1141	1259	0.0160
Brindle	Brindle	Black brindle	110	148	0.0180
Fawn	Black brindle	Brindle	63	91	0.0241
Brindle	Black	Black	488	560	0.0261
Black	Grey	Black	159	198	0.0390
Black	Brindle	Black brindle	61	86	0.0392

For brindle offspring, when brindle males were crossed with grey brindle females, 90 males and 55 females were produced. This result did not confirm a ratio of 1:1 using the chi-square test (*p* = 0.0037).

For black offspring, when black males were crossed with brindle females, 524 males and 615 females were produced. This result did not confirm a ratio of 1:1 using the chi-square test (*p* = 0.007). A ratio of 1:1 was not confirmed in an additional 6 offspring colour groups using statistically significant data (Table 1).

## Discussion

We found that crossing black-, fawn- and grey-colour individuals (male and female of the same colour) resulted in a greater than 70% probability that offspring would present the same colour as their parents. On the other hand, the coat colour of offspring arising from crossing individuals of black brindle, grey brindle and brindle was much more dependent on the genetic background. Crossing parents of the same colours can produce a litter with multiple colour variations. This is an important finding because there is a demand for specific colours within the community of breeders.

The results of sex segregation ratios confirmed our hypothesis that at least one gene responsible for coat colour is located on a sex chromosome. It is possible that one of the control mechanisms or biochemical pathways controlling the expression of coat colour could be regulated by sex chromosomes. Our hypothesis is supported by the fact that a gene located on a sex chromosome determines orange colouring in cats. This gene is still unknown, but its effect on coat colouring has been known for decades [1, 28].

In dog coat colour genetics, there are many unknown mechanisms that lead to the expression of some colour phenotypes other than the basic colours (for example, ticking, progressive greying and tweed phenotypes). Hypothetically, the intensity of fawn, red, brindle or grey colours could be affected by gene or genes that are still unknown.

## Conclusions

We are the first to describe coat colour segregation ratios in Cane Corso Italiano dogs. Segregation ratios that do not follow a Mendelian pattern indicate that additional genes are active in the determination of coat colour. For the first time, we described that at least one gene responsible for a dog’s coat colour is located on a sex chromosome. The genetics of coat colour in dogs is a very progressive research field and a popular scientific topic.

## Abbreviations

ASIP: Agouti signalling protein
CBD103: Canine *beta*-defensin 103
FCI: Federation Cynologique Internationale
MC1R: Melanocortin 1 receptor
